# Factors influencing mothers’ decisions regarding obstetrical care in Western Kenya: a mixed-methods study

**DOI:** 10.1186/s12905-021-01355-9

**Published:** 2021-05-19

**Authors:** Grace Umutesi, Matthew D. McEvoy, Kemberlee Bonnet, Sophie Druffner, David G. Schlundt, Harrysone E. Atieli, Joy N. China, Kennedy Onyango, Mark W. Newton

**Affiliations:** 1 Department of Anesthesiology, Vanderbilt University Medical Center, 2301 Vanderbilt University Hospital, Nashville, TN 37232-7237 USA; 2 Department of Psychology, Vanderbilt University, 111 21st Ave S, Nashville, TN 37240 USA; 3 Peabody College, Vanderbilt University, 230 Appleton Pl #5721, Nashville, TN 37203 USA; 4 Maseno University School of Medicine, Kondele Kisumu-Busia Rd Maseno, Kisumu, Kenya; 5 Department of Anesthesiology, AIC Kijabe Hospital, Kijabe Road, Kijabe, Lari, Kiambu Kenya; 6 Vanderbilt University Medical Center, 2200 Children’s Way, Suite 3115, Nashville, TN 37232-9070 USA

**Keywords:** Three Delays Model, Maternal mortality, Decision-making, Healthcare seeking behavior, Low-income countries, Kenya

## Abstract

**Background:**

Siaya County in Western Kenya has one of the highest maternal mortality rates in Kenya. We sought to elucidate factors that influence mothers’ decisions regarding where to seek obstetrical care, to inform interventions that seek to promote effective use of obstetric services and reduce maternal mortalities. To guide our research, we used the “Three Delays Model”, focusing on the first delay—seeking care. While interventions to reduce maternal mortalities have focused on addressing delays in accessing and receiving care, context-specific data on drivers of the first delay are scarce.

**Methods:**

We used a mixed-method study to assess how maternal decision-making of birth location is influenced by personal, contextual, and cultural factors. We conducted structured interviews with women aged 14 years or older living in Siaya, Bondo, and Yala, rural districts in Western Kenya. We then conducted focus group interviews with a subset of women to elucidate this question: How do drivers of the first delay (i.e., seeking care) affect the decision to seek home versus hospital delivery, potentially negatively influencing maternal mortality.

**Results:**

Three hundred and seven women responded to the surveys, and 67 women (22%) from this group participated in focus group interviews. Although we focused on type 1 delays, we discovered that several factors that impact type 2 and type 3 delays directly contribute to type 1 delays. Our findings highlighted that factors influencing women’s decisions to seek care are not simply medical or cultural but rather contextual, involving many elements of life, particularly in rural communities.

**Conclusions:**

It is imperative to address multiple-level factors that influence women’s decisions to seek care and have in-hospital deliveries. To curtail maternal mortality in rural Western Kenya and comparable settings, targeted interventions must take into consideration these important influencers.

**Supplementary Information:**

The online version contains supplementary material available at 10.1186/s12905-021-01355-9.

## Background

In 2015, 99% of maternal mortalities occurred in developing countries [1, 2]. More than half of these deaths took place in sub-Saharan African countries, where the estimated maternal mortality rate is as high as 546 maternal deaths per 100,000 live births [3, 4]. Healthcare seeking behavior often affects health outcomes, such as when a mother requiring medical attention delays seeking or accessing care. Training healthcare providers and equipping systems with appropriate infrastructure are important steps to achieving high quality care [5]. Additionally, maternal health services must be safe, effective, efficient, timely, equitable, and people-centered to improve obstetric outcome [6]. Access to a high quality of care is essential to achieving better health outcomes; however, if the factors that influence healthcare seeking behavior are not addressed, obstetric services might not be used to their full potential.

In an attempt to delineate factors influencing maternal mortality in low- and middle-income countries, the “Three Delays Model” was created to highlight the main causes of pregnancy-related mortality in Haiti [7]. This framework stated that pregnancy-related mortality is due to delays in (1) seeking care, (2) reaching care, and (3) receiving adequate care once at the facility. In recent years, many programs have targeted the second and third delays in health systems. However, an understanding of individual and health system drivers of the first delay is essential to creating programs that will effectively decrease pregnancy-related deaths [5, 8, 9].

Previous studies have looked at the role of systems in preventing pregnancy-related deaths in low- and middle-income countries, but assessments of “patient factors” that influence where women choose to deliver are either scarce or outdated [5, 10]. Comprehensive studies on maternal mortality and morbidity are limited, and they often report findings from data collected in hospital settings, thus excluding women who do not seek hospital-based care [11]. In Kenya (population 47 million), 62.6% of women deliver at healthcare facilities, and the maternal mortality rate (MMR) is as high as 362 maternal deaths per 100,000 live births [12–14]. Western Kenya accounts for a large portion of maternal mortalities in Kenya, and a woman who opts for home delivery without a trained healthcare provider is 3 times more likely to experience perinatal death than one who gives birth at a healthcare facility [15].

An understanding of the patient perspective is imperative to promote the effective use of obstetric services. We used a mixed-method study to assess how maternal decision-making of birth location is influenced by personal, contextual, and cultural factors.

## Methods

### Ethical considerations

Ethics approval for this study was obtained from Vanderbilt University Medical Center (Nashville, TN, USA) and Maseno University (Kisumu, Kenya). Additionally, approvals were obtained from Siaya County (Kenya) and the hospital leadership offices at each participating site. Written informed consents were obtained from study participants prior to enrollment.

### Study site/participants

Yala, Bondo, and Siaya Districts are located in Siaya County in Western Kenya with a total population of approximately 984,069 [16]. Quantitative and qualitative data were collected in September 2016 to assess women’s perception of obstetric care in rural Western Kenya. Participants were recruited within the catchment area of Yala, Bondo, and Siaya referral hospitals. Inclusion criteria consisted of (1) being 14 years or older; (2) living within the catchment areas of the 3 referral hospitals; and (3) having had at least 1 pregnancy. Participants were compensated with a prepaid phone card, and those who participated in the focus group interviews were offered refreshments during the sessions.

### Procedure and data collection tools

The recruitment occurred at 3 referral hospitals and in the surrounding areas within a 5-mile radius from each hospital (e.g., at markets, shops), where participants were randomly selected. If participants were interested in voluntarily participating in the study and met the inclusion criteria, a study staff member obtained written informed consent. The trained facilitator, fluent in Luo and Swahili, facilitated the recruitment process and allowed study participants to participate in their primary language. The trained facilitator then conducted structured interviews, and the questionnaires were available in English, Swahili, and Luo. Structured interviews were conducted to capture demographic, past pregnancy experiences, and patient satisfaction data (Additional file 1: Appendix 1, Interview Questionnaire [supplemental digital content]). All quantitative data from structured interviews were de-identified and entered by the interviewer into the Research Electronic Data Capture (REDCap) mobile application [17].

After completing the in-depth interviews, participants were randomly selected and invited to participate in focus group interviews. A separate consent was obtained and interviews were conducted by a trained data collector who was the focus group moderator (JC). Focus group sessions were conducted in English, Swahili, or Luo, depending on the language preferred by the participants. All focus group responses were de-identified, and they were recorded using participant identification numbers to keep the participants anonymous and protect their privacy. Open-ended scripted questions were asked using a moderator’s guide that included questions pertaining to number of pregnancies in the community, maternity services, and community perceptions of hospital and traditional birth attendant (TBA) delivery. Additionally, these questions captured information regarding factors that influenced a mother’s choice on where and how to deliver, perceptions of cesarean delivery (C-section), and logistical factors related to traveling to medical facilities (Additional file 2: Appendix 2, Community Focus Group Discussion [supplemental digital content]). Follow-up questions were asked for clarity and to acquire additional data. Audio recordings were translated into English and transcribed by the moderator (JC), who is fluent in all 3 languages.

### Analysis

Demographic characteristics were compiled from descriptive statistics using Stata Statistical Software Version 14.2 (StataCorp LP, College Station, Texas). Data coding, analysis, and reporting were completed by following the Consolidated Criteria for Reporting Qualitative Research (COREQ) guidelines, an evidence-based qualitative methodology [18]. Transcribed focus group transcripts were imported into Microsoft Excel files for coding. An inductive-deductive approach was used to develop the coding system and create the conceptual framework based on the moderator’s guide questions and a preliminary review of 3 focus group transcripts. The coding system was organized into major categories and then subcategorized to capture further thematic detail. Categories captured main themes of (1) community perceptions of hospital delivery; (2) concerns with hospital delivery; (3) perceptions of traditional birth attendants; (4) perceptions of cesarean delivery versus vaginal delivery; (5) costs related to care; and (6) transportation to a healthcare facility. Each of these major categories was divided into 2 to 9 subcategories. Specific definitions were written for each category, along with coding rules for using each category.

Each statement within the transcript was treated as a separate quote, and each quote could be assigned up to 5 different codes. Two trained coders independently coded the quotes and established consensus to resolve differences in coding. Quotes were sorted by category, frequency distributions were calculated, and then quotes were reviewed to identify higher-order themes. The analysis was guided by Social Ecological Theory, and the purpose of the analysis was to develop the explanatory framework that best described factors at the individual, community, and health systems levels that contribute to participants’ perceptions of traditional delivery and hospital delivery [19].

## Results

Three hundred and eight women participated in the structured interviews, and 67 (21.8%) of these engaged in focus group interviews. Responses from one participant were excluded from the analysis since she lived outside the catchment area of the three referral hospitals. The overall median age was 26 years (IQR 23–32). Overall, the majority of participants were married (234 [76.2%]) with variability across sites mainly in Siaya where married women constituted 65.7% of study participants and over a third of study participants were single. Moreover, 99% of participants had completed at least a primary education, equivalent to the sixth grade in the United States education system, with a median parity of 2 (IQR 1–3) (Table 1).

**Table 1:** Demographic characteristics of respondents

	Yala (n = 97)	Bondo^b^ (n = 102)	Siaya (n = 108)	Total (N = 307)
Age^a^	28 (23–35)	26 ( 23–32)	26 (22–31)	26 (23–32)
Catchment	21,999	42,506	44,195	108,700^c^
Marital status [n (%)]				
Married	78 (80.4)	85 (83.3)	71 (65.7)	234 (76.2)
Single	15 (15.5)	13 (12.8)	34 (31.5)	62 (20.2)
Divorced	2 (2.1)	1 (0.98)	1 (0.9)	4 (1.3)
Separated	0	2 (1.96)	1 (0.9)	3 (0.98)
Widowed	2 (2.1)	1 (0.98)	1 (0.9)	4 (1.3)
Parity	2 (1–3)	2 (1–3)	1 (1–3)	2 (1–3)
Hospital deliveries [n (%)]	91 (93.8)	93 (91.1)	97 (89.8)	281 (91.5)^d^
Education (≥ Primary education) [n (%)]	96 (98.97)	101 (99)	107 (99.1)	304 (99)
Focus group participants [n (%)]	26 (26.8)	21 (20.6)	20 (18.5)	67 (21.8)

^a^Continuous variables presented as median (IQR)

^b^1 participant resident from Ugunja was excluded from the analysis. In 2012, the Maternal Mortality Rate in Siaya was 117.1 maternal deaths per 100,000 live births. (Republic of Kenya Ministry of Health. Health Sector Human Resources Strategy 2014–2018. Available at: http://www.health.go.ke/wp-content/uploads/2016/04/Kenya-HRH-Strategy-2014-2018.pdf. Accessed January 8, 2019.)

^c^The total expected population for Siaya County in 2015 was 932,795. (Republic of Kenya, County Government of Siaya. Medium Term: Annual Development Plan 2017–2018. Available at: http://siaya.go.ke/wp-content/uploads/2017/09/SIAYA-COUNTY-2017-18-ADP-FINAL.pdf. Accessed January 8, 2019.)

^d^69.6% of women 15–49 years delivered in a health facility in Siaya County in 2014 [14]

**Fig. 1:**
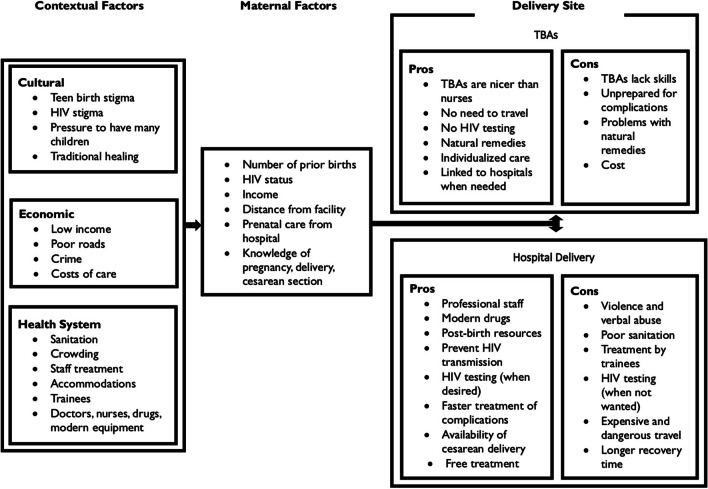
Matrix of forces directly affecting women and influencing their perceptions of traditional birth attendants (TBAs) and hospital delivery

Using an iterative inductive-deductive process based on the coding system, frequency of codes, and a review of the quotes sorted by category, a framework was developed to highlight the specific contextual and maternal factors that influence perception and decision to deliver at home with a traditional birth attendant or at the hospital. Figure 1 identifies culture, economic influences, and the health system as key contextual factors directly affecting the mother’s decision tree regarding location of delivery while highlighting the perceived advantages and disadvantages of traditional delivery at home versus hospital delivery.

### Contextual factors

We identified culture, economic influences, and the health system as the 3 primary contextual factors influencing mothers’ decisions about birth location.

1. Cultural factors

Women discussed influential pregnancy-related cultural factors including both teen birth stigma and the pressure to have many children. Younger women reported experiencing additional stress while seeking to hide their pregnancies or consider abortion. These women also tended to prefer a home delivery due to the stigma noted in the hospital setting.

“Most pregnant mothers are teenagers and they hide the pregnancy until during birth where they deliver at home or on the roads or attempt abortions because they fear been known [sic] by parents.” Participant 5, FG5, Yala

“Due to the increased early pregnancies in Siaya as a whole, some would fear hospitals, as they are young and not everyone will perceive them well…hence many will prefer home deliveries…” Participant 5, FG4, Siaya

In Western Kenya, having many children contributes positively to a mother’s social status, although this reality was expressed indirectly. This factor also led women to favor home deliveries, as many women associate hospital delivery with cesarean delivery, which could both limit future pregnancies and result in an extended recovery time.

“It (C-section) limits the number of pregnancies they can have. I hear you have to wait for a few years before trying to get pregnant again.” Participant 5, FG1, Siaya

2. Economic factors

The dominant themes pertaining to economic factors were low income, hospital costs, poor roads, and concerns about safety. Mothers with limited income feared not being able to pay for hospital procedures and safe transportation.

“Some can only get 100 KSH [~ $1] a day and not guaranteed to get the same amount daily. Most people do not even have an income.” Participant 3, FG3, Yala

“For an operation, they pay about 1,000 KSH [$10] for which most mothers cannot afford because they are unemployed.” Participant 4, FG1, Bondo

Poor road conditions were also noted as a barrier to hospital delivery. Poor road conditions slowed travel and often resulted in a mother delivering on the side of the road.

“The roads are not proper in all areas. There are places people have to walk. It’s rocky…they deliver on the road and end up calling people from homesteads for help (sighs).” Participant 1, FG2, Yala

Crime during transit was another perceived problem associated with travel to the hospital for delivery.

“When there is fully grown maize in the plantations, insecurity is high because robbers stand around the roads.” Participant 3, FG4, Yala

3. Health system factors

Additionally, women reported that health system factors directly influenced their decisions regarding where to give birth. These include hospital sanitation, crowding, patient treatment by staff, the role of medical trainees, the availability of modernized care, and inadequate feminine hygiene products.

“Improve the hygiene especially the beddings, delivery rooms, and kitchen conditions…the kitchen is just next to the morgue! (sighs)…and flies move from the morgue to the kitchen.” Participant 3, FG3, Siaya

Women reported that wards were so crowded that women were forced to share beds, pre- and postpartum, at times sharing the obstetrical ward with general medical patients.

“They sleep two or more in bed with their newborns and also share the ward with pediatric admissions.” Participant 4, FG5, Yala

Poor treatment by staff was common. Some women communicated a fear of staff based upon community news that mothers had died as a result of hospital-based patient abuse.

“Nurses who attend to them at the hospital are very harsh and they also handle them badly. There are rumors of those who die when delivering at the hospitals.” Participant 3, FG 4, Siaya

Participants noted other problems when discussing the hospital environment, such as poor food quality, lack of clean water, and unstable electricity on the wards without backup generators.

Positive elements of the healthcare system mentioned were trained professional staff, the availability of medicines, and modern equipment.

“Now, there is an increase in the number of doctors and surgeons during operation, and also now there is new machine that have made the operations easier.” Participant 5, FG3, Siaya

### Maternal factors

Maternal factors that affected the decision to have a home or hospital delivery included distance from a facility, HIV status, and past experience with prenatal hospital care. Women in remote areas were often not educated on the benefits of delivering at a hospital. Some women who used traditional birth attendants had negative feelings about hospitals or were influenced by others’ negative attitudes about hospitals.

“Others just have a negative attitude and feel that there is nothing to gain in hospital. Some people are told that hospitals are not good.” Participant 5, FG2, Bondo

“You will find people still seeking services of the traditional birth attendants due to lack of awareness. This is a rural area; hence people are not informed.” Participant 2, FG4, Siaya

Geographic distance from a healthcare facility influenced women’s choice of where to deliver. Women living in remote areas far from the main roads or hospital often used traditional birth attendants (TBAs) because they did not have transportation funds or access to vehicles enabling travel to hospitals.

“TBAs are close to homes of people, and the hospitals are quite far, so it is the only option pregnant mothers have.” Participant 5, FG5, Yala

HIV status alone, if positive or not tested, was also found to be an important maternal factor. Mothers did not want to be tested at the hospital or stigmatized about their positive status, so they preferred a home delivery.

“There are those mothers who fear going to the hospital because they fear being tested for HIV and do not want to know their status.” Participant 3, FG5, Yala

“The TBAs are preferred because they do not test for HIV like in the hospital.” Participant 3, FG1, Bondo

Prenatal care provided by the hospital had a critical influence on a woman’s choice to have a hospital delivery. Women who did not obtain prenatal care from the hospital were often rejected by the hospital at the time of delivery. Nurses refused to provide care to women who did not receive prenatal care, and these women reported having to delivery without medical assistance even in the waiting room.

“If you did not attend the antenatal clinics, then you come to the hospital in labor, the nurses will not attend to you. They let you deliver by yourself in the waiting area. Hence you will find many women shunning away from the hospital if they did not attend clinics during pregnancy.” Participant 2, FG2, Siaya

### Delivery location

#### Home delivery

TBAs were also viewed as being more attentive with personalized care since most hospital nurses were taking care of several laboring patients.

“The nurses in the hospital serve so many women, like about 20, and get exhausted, which make them harass mothers, but TBAs are good because they have only one mother at a time, which allows them to give full attention to mothers.” Participant 2, FG3, Bondo

TBAs come to a woman’s home, eliminating the need for the mother to travel to a hospital which adds to the cost as well as security issues.

“At times labor comes at night, and no one will take you to hospital late at night so they call on TBAs who are near the homes of these pregnant mothers.” Participant 2, FG3, Bondo

Additionally, TBAs’ practice of providing herbal remedies during birth was seen as a positive benefit since hospital nurses cannot use herbal drugs.

“These days TBAs have herbal treatment which when given they can change a baby's position if it is not correct, unlike in a hospital, where they do not have drugs to make the baby turn around in the stomach and the doctors will not know.” Participant 3, FG3, Bondo

Some feared that a TBA’s lack of skills could result in having to be transferred to a hospital during birth, particularly in the event of a medical emergency.

“They can deliver the baby badly and the baby can die, or they may cut the cord in a bad way and that may cause a lot of problems, and also some bleed a lot and they have to be rushed to the hospital.” Participant 5, FG1, Siaya

TBAs were also seen as having a known cost associated with their service, whereas hospital vaginal deliveries are free but the cost could increase with complications.

“People also don’t prefer them (TBAs) because they will ask you for money.” Participant 1, FG1, Siaya

#### Hospital delivery

Hospitals were seen as having recently improved, especially in their ability to provide emergency treatment, deal with complications, and perform cesarean deliveries.

“Availability of drugs at the hospital to prevent infections during operation. In hospitals you find doctors and nurses who are here to help you in case of any problem.” Participant 1, FG1, Yala

Hospitals were also preferred because post-birth resources such as basins, diapers, and medications were availability. Ascertainment of HIV status and prevention of HIV transmission to the baby through use of medications administered during labor were seen by some women as a benefit of hospital birth.

“In hospital it is better because you are tested for HIV and if you are positive the baby will not get the virus at the hospital. If you go to the TBAs they will not test you for HIV.” Participant 4, FG3, Bondo

Reasons for not choosing a hospital birth included staff abuse, lack of sanitation, the cost of cesarean delivery, and travel burden. The role of student trainees was seen as a reason to avoid hospital births. Women reported unsupervised trainees performing cesarean delivery.

“These days, SVD (spontaneous vaginal delivery) is better, as most operations here are performed by students and interns who are not fully qualified. Someone was cut inappropriately through CS by a student.” Participant 5, FG4, Siaya

## Discussion

### Main findings

In Western Kenya, as in many low-resource settings, a bias in favor of home birth over hospital delivery remains—influenced by complex and interrelated factors [13, 20]. Furthermore, home delivery without trained health providers has been shown to increase perinatal mortality threefold [21]. In response to the 2010 United Nations report *Global Strategy for Women's and Children's Health*, the Government of Kenya operationalized 210 primary health facility centers of excellence to provide maternal and child health services to an additional 1.5 million women and 1.5 million children [11]. Such governmental efforts were intended to address financial hurdles for maternal and child care. The literature, however, has documented that improving access to healthcare services does not assure the use of those services [22, 23]. This study highlights factors that contribute to the 3 types of delay that prevent women from receiving maternal care within a medical care facility. Indeed, in a low-resource setting, a woman’s “choice” of where to seek care—at home or the hospital—is influenced primarily by factors beyond her control.

### Seeking care—Type 1 delay factors

In Siaya County, a number of factors influence the use of hospital-based obstetric services, and these go well beyond the mere availability of advanced hospital training and modern equipment. Importantly, we found that past experiences influence future care decisions. A woman who experienced substandard quality of care at a healthcare facility in the past was less likely to seek hospital-based care for subsequent pregnancies (type 1 delay) [2, 23, 24]. Similarly, having received individualized care from a traditional birth attendant swayed women toward a subsequent home birth. Moreover, a study done in Kilifi county stated that older women who have more experience and have previously delivered without any complication tend to deliver at home [25]. Our findings also indicate that women in Siaya County fear the consequences of having a cesarean delivery. Data from the 2014 demographic survey in Kenya revealed that more than 83% of women who have cesarean birth stay in the hospital for at least 3 days after giving birth [14]. Our results provided evidence that women’s fear of a long recovery post cesarean delivery contributes to the type 1 delay.

Additionally, in many parts of Africa, women suffer serious social repercussions because of their HIV status [26]. HIV testing services in hospitals are advantageous but may expose mothers to negative social consequences, including community stigmatization. In our study, a stated fear of stigmatization correlated with a preference for home birth. Programs that seek to improve maternal and neonatal health should invest in increasing patients’ confidence in hospital confidentiality practices, especially regarding HIV status. This hospital culture shift should include staff sensitization and professionalism seminars, with plans for disciplinary action when confidentiality is not practiced.

### Reaching the Hospital—Type 2 delay factors

Among our focus group participants, real or perceived difficulties related to traveling to the hospital negatively affected the decision to seek care away from home (type 2 delay). Some women were concerned about being harassed, or even becoming a victim of crime, on their way to the healthcare facility, which influenced their decision even more than concerns related to the cost of travel. Type 2 delays certainly could improve with expensive and prolonged road projects, but such projects will impact only those within the reach of these road systems. One potential solution would be “waiting homes,” residential facilities located near qualified hospitals to which women in rural areas could travel in the final weeks of pregnancy, to ease the burden and danger of traveling just before birth [25, 27]. Certainly, such homes have cultural and financial implications; nevertheless, practical solutions must be attempted.

### Receiving adequate care—Type 3 delay factors

Our study shows that women are concerned with hospital sanitation, care systems, and staff professionalism. Raising standards for sanitary conditions would improve the experience of mothers at hospitals. The presence of trained healthcare providers was also mentioned as a factor that directs the choice of where women deliver. Having qualified personnel who can provide safe anesthesia and surgery care with systems in place to reduce the risk of postpartum complications, such as infections, could prompt mothers to seek care without delay. Sadly, our focus group participants also raised concerns about being harassed by healthcare providers in facilities. Robust professionalism training should be delivered in these facilities to ensure that healthcare providers are trained on the importance of patient respect, as other initiatives seek to address other existing hurdles (e.g., infrastructure, supplies, medication, and awareness).

The limitations of our study include recall bias for participants whose last pregnancy occurred long before the interviews. Some participants may have shared stories of births that occurred in other counties, before moving to Siaya, although the impact of this on results would be minimal. Additionally, we sought to address selection bias by randomly recruiting participants from both the referral hospitals and the community within a 5-km radius from the hospital. Desirability bias could have been a limitation of responses provided, but we noted that there was a wide variety of insights shared by study participants during focus group interviews. Due to time limitation, as well as resources, the study team was not able to travel beyond this defined area to enroll additional study participants. The majority of study participants had delivered in healthcare facilities, thus limiting our ability to capture a considerable number of women who had delivered at home. These data, despite not being representative of the situation at the national level, present perceptions from women in different geographical areas (urban, semi-urban, and rural) in Siaya County.

## Conclusions

Our findings highlight factors that delay mothers from seeking care (type 1 delay) within one region of Western Kenya. Although we focused on type 1 delays, we discovered that several factors that impact type 2 and type 3 delays also directly contribute to type 1 delays. This study demonstrates that maternal delays in care are not a continuum but a constant feedback loop where type 3 delays positively or negatively impact type 1 delays. Factors contributing to maternal-care–seeking behavior are not simply medical or cultural. Rather, decisions are contextual, involving many elements of life, particularly in rural communities. Home births are associated with higher risks of pregnancy-related mortalities, and factors that hinder women from delivering at healthcare facilities should be addressed in order to improve outcomes. Ultimately, mothers must weigh many options when seeking maternal care, and they need to be assisted with a practical and cost-efficient approach so that maternal and newborn health outcomes improve. Our future studies will investigate educational interventions focusing on type 1 delays based upon the patient level data obtained from this study.

## Supplementary Information

**Additional file 1: Appendix 1**. Interview questionnaire. This tool was used to capture in-depth data from study participants.

**Additional file 2: Appendix 2**. Focus group guide. This tool, containing open-ended scripted questions, was used by the focus group moderator to help guide the focus group interviews and discussion. The focus group sessions were held in English, Swahili, or Luo, depending on the language preferred by the participants.

## Data Availability

The datasets analyzed during the current study are available from the corresponding author on reasonable request.

## Abbreviations

TBA: Traditional birth attendant
C-section, CS: Cesarean delivery
COREQ: Consolidated Criteria for Reporting Qualitative Research
FG: Focus group
SVD: Spontaneous vaginal delivery

## References

[1] World Health Organization. Trends in Maternal Mortality: 1990 to 2015: Estimates by WHO, UNICEF, UNFPA, World Bank Group and the United Nations Population Division. Geneva: World Health Organization; 2015. http://www.afro.who.int/sites/default/files/2017-05/trends-in-maternal-mortality-1990-to-2015.pdf. Accessed 8 January 2019.

[2] Mgawadere F, Unkels R, Kazembe A, van den Broek N (2017). Factors associated with maternal mortality in Malawi: application of the three delays model. BMC Pregnancy Childbirth.

[3] Alkema L, Chou D, Hogan D (2016). Global, regional, and national levels and trends in maternal mortality between 1990 and 2015, with scenario-based projections to 2030: a systematic analysis by the UN Maternal Mortality Estimation Inter-Agency Group. Lancet.

[4] World Health Organization. Maternal Mortality: Key Facts. 2018 Feb. http://www.who.int/news-room/fact-sheets/detail/maternal-mortality. Accessed 8 Jan 2019.

[5] Girum T, Wasie A (2017). Correlates of maternal mortality in developing countries: an ecological study in 82 countries. Matern Health Neonatol Perinatol.

[6] Maternal Health Task Force. Quality of Maternal Health Care. https://www.mhtf.org/topics/quality-of-maternal-health-care/. Accessed 8 Jan 2019.

[7] Barnes-Josiah D, Myntti C, Augustin A (1998). The, "three delays" as a framework for examining maternal mortality in Haiti. Soc Sci Med.

[8] Kea AZ, Tulloch O, Datiko DG, Theobald S, Kok MC (2018). Exploring barriers to the use of formal maternal health services and priority areas for action in Sidama zone, southern Ethiopia. BMC Pregnancy Childbirth.

[9] Pacagnella RC, Cecatti JG, Osis MJ, Souza JP (2012). The role of delays in severe maternal morbidity and mortality: expanding the conceptual framework. Reprod Health Matters.

[10] Sundari TK (1992). The untold story: how the health care systems in developing countries contribute to maternal mortality. Int J Health Serv.

[11] Filippi V, Chou D, Ronsmans C, Graham W, Say L. Levels and causes of maternal mortality and morbidity. In: Black RE, Laxminarayan R, Temmerman M, Walker N, eds. Reproductive, Maternal, Newborn, and Child Health: Disease Control Priorities. 3rd ed. (volume 2). Washington (DC): The International Bank for Reconstruction and Development / The World Bank; 2016. Chapter 3.

[12] Central Intelligence Agency. The World Factbook: Kenya. https://www.cia.gov/library/publications/the-world-factbook/geos/ke.html. Accessed 8 Jan 2019.

[13] The Partnership for Maternal, Newborn and Child Health. Maternal and Child Health: Kenya. http://www.who.int/pmnch/media/membernews/2011/20121216_kenyaparliament.pdf. Accessed 8 Jan 2019.

[14] Kenya National Bureau of Statistics. Kenya Demographic and Health Survey 2014. https://dhsprogram.com/pubs/pdf/FR308/FR308.pdf. Accessed 8 Jan 2019.

[15] Gabrysch S, Campbell OM (2009). Still too far to walk: literature review of the determinants of delivery service use. BMC Pregnancy Childbirth.

[16] Ministry of Health. Siaya County, Health at a Glance. May 2015. https://www.healthpolicyproject.com/pubs/291/Siaya%20County-FINAL.pdf. Accessed 8 Jan 2019.

[17] Harris PA, Taylor R, Thielke R, Payne J, Gonzalez N, Conde JG (2009). Research electronic data capture (REDCap)–a metadata-driven methodology and workflow process for providing translational research informatics support. J Biomed Inform.

[18] Tong A, Sainsbury P, Craig J (2007). Consolidated criteria for reporting qualitative research (COREQ): a 32-item checklist for interviews and focus groups. Int J Qual Health Care.

[19] Stokols D (1992). Establishing and maintaining healthy environments. Toward a social ecology of health promotion. Am Psychol..

[20] Sharma SR, Poudyal AK, Devkota BM, Singh S (2014). Factors associated with place of delivery in rural Nepal. BMC Public Health.

[21] Mrisho M, Schellenberg JA, Mushi AK (2007). Factors affecting home delivery in rural Tanzania. Trop Med Int Health.

[22] Ensor T, Cooper S (2004). Overcoming barriers to health service access: influencing the demand side. Health Policy Plan.

[23] Thaddeus S, Maine D (1994). Too far to walk: maternal mortality in context. Soc Sci Med.

[24] van den Broek NR, Graham WJ (2009). Quality of care for maternal and newborn health: the neglected agenda. BJOG.

[25] Moindi RO, Ngari MM, Nyambati VC, Mbakaya C (2016). Why mothers still deliver at home: understanding factors associated with home deliveries and cultural practices in rural coastal Kenya, a cross-section study. BMC Public Health.

[26] Rankin WW, Brennan S, Schell E, Laviwa J, Rankin SH (2005). The stigma of being HIV-positive in Africa. PLoS Med.

[27] World Health Organization. Maternity waiting homes: a review of experiences. 1996. https://apps.who.int/iris/bitstream/handle/10665/63432/WHO_RHT_MSM_96.21.pdf;jsessionid=D398A6AF16B932AC7130F8FDE1202A57?sequence=1. Accessed 22 July 2019.

