# Supplementary material for: Factors influencing mothers’ decisions regarding obstetrical care in Western Kenya: a mixed-methods study
Source: BMC Womens Health. 2021 May 19;21:210. doi: 10.1186/s12905-021-01355-9 (PMC8136230; doi:10.1186/s12905-021-01355-9)
Supplement: Supplementary file 1 — Additional file 1: Appendix 1. Interview questionnaire. This tool was used to capture in-depth data from study participants. [file 12905_2021_1355_MOESM1_ESM.pdf]

Interview Questionnaire

Record ID	______________________
Date	______________________
Participant ID	______________________<br>(K(B, S or Y) 00 #/C/(Month)16)
1. What is your age?	______________________
2. What is your marital status?	◯ Single<br>◯ Married<br>◯ Divorced<br>◯ Separated<br>◯ Widowed
3. Which district do you live in?	◯ Bondo<br>◯ Siaya district<br>◯ Yala<br>◯ Other
3. a. Specify:	______________________
4. Did you attend formal school?	◯ Yes<br>◯ No
4. a. If yes, what is your highest completed level of education?	◯ Primary<br>◯ Some secondary<br>◯ Completed secondary<br>◯ College/ University<br>◯ Graduate level (Masters/ PhD)

I would like to ask you questions regarding the most recent pregnancy and previous pregnancies:

5. How many pregnancies, including the past one, have you had?	______________________
5. a. How many were born at home?	______________________
5. b. How many were born at the hospital?	______________________
6. When was your most recent pregnancy?	______________________<br>(Precise if it is the number of years, months or days since your last pregnancy)

Where did you give birth?

	Home	Health Facility	Other
7. For your last pregnancy:	◯	◯	◯
8. For the next to last pregnancy:	◯	◯	◯

8. a. Specify

____
(Specify for both pregnancies if needed)

If you delivered at home, what was the cause cause? Select all that apply

	Personal choice	Lack of transportation	Could not afford it	Other
9. For the last pregnancy	☐	☐	☐	☐
10. For the next to last pregnancy	☐	☐	☐	☐

10. a. Specify:

____
(Specify for both pregnancies if needed)

What was the reason for you to go to a health facility? Select all that apply

	I came on my own	I was referred from a lower level health facility	I had complications like bleeding	The baby would not come out	I had headaches	Other
11. For the last pregnancy:	☐	☐	☐	☐	☐	☐
12. For the next to last pregnancy:	☐	☐	☐	☐	☐	☐

12. a. Specify:

____
(Specify for both pregnancies if needed)

If you were referred, which facility were you referred FROM?

	Government dispensary	Government Sub-County	County Referral	Private nursing home or hospita
13. For the last pregnancy	◯	◯	◯	◯
14. For the next to the last pregnancy	◯	◯	◯	◯

If you were referred, which facility were you referred TO?

	Government dispensary	Government Sub-County	County Referral	Private nursing or hospital
15. For the last Pregnancy	◯	◯	◯	◯

16. For the next to the last Pregancy	○	○	○	○

How did you get to the health facility?

	Walk	Motor vehicle	Cart/ Bicycle/ tricycle	Boat	Ambulance (motorized or bicycle)	Other
17. For the last pregnancy:	○	○	○	○	○	○
18. For the next to the last pregnancy	○	○	○	○	○	○

18. a. Specify: ______________________________
(Specify for both pregnancies if needed)

Which kind was the transportation?

	Public	Private	Not Applicable
19. For the last pregnancy:	○	○	○
20. For the next to the last pregnancy	○	○	○

21. How much did the transportation cost(Ksh) for the last pregnancy? ______________________________

22. How much did the transportation cost(Ksh) for the next to the last pregnancy? ______________________________

How long did it take you to get to the facility?

	5 - 29 min	30 - 59 min	60 - 89 min	90 - 119 min	More than 2hrs (120min)
23. For the last pregnancy:	○	○	○	○	○
24. For the next to the last pregnancy:	○	○	○	○	○

How far was the health facility

	Less than 1 km	1 - 4 km	5 - 9 km	10 - 15 km	More than 15 km
25. For the last pregnancy:	○	○	○	○	○
26. For the next to the last pregnancy:	○	○	○	○	○

How did you deliver?

	Vaginal	C-section
27. For the last pregnancy:	○	○
28. For the next to the last pregnancy:	○	○

If you had a c-section; did you have anesthesia?

	Yes	No
29. For the last pregnancy:	○	○
30. For the next to the last pregnancy:	○	○

Was the baby preterm?

	Yes	No
31. For the last pregnancy:	○	○
32. For the next to the last pregnancy:	○	○

33. At what gestation stage did you deliver for the last pregnancy? ______________________

34. At what gestation stage did you deliver for the next to last pregnancy? ______________________

What was the condition of the baby delivered?

	Alive	Stillbirth	Born- died within 48hrs
35. For the last pregnancy:	○	○	○
36. For the next to last pregnancy	○	○	○

37. What was the weight of the baby at birth for the last pregnancy? ______________________
(Precise if it is in Kg or lbs)

38. What was the weight of the baby at birth for the next to the last pregnancy? ______________________
(Precise if it is in Kg or lbs)

If the baby died after birth; what was the cause of the newborn's death?

	Preterm	Fever	Breathing difficulty	Yellow eyes/ skin (Jaundice)	Other	Not applicable
39. For the last pregnancy:	○	○	○	○	○	○
40. For the next to the last pregnancy:	○	○	○	○	○	○

Specify: ______________________
(Specify for both pregnancies if applicable)

If the baby died after birth, what was the location of the newborn's death

	Home	Hospital	Other
41. For the last pregnancy:	○	○	○
42. For the next to the last pregnancy:	○	○	○

Specify: ______________________

43. How much in total did you pay for the obstetric procedure for your last pregnancy? ______________________ (Ksh)

44. How much in total did you pay for the obstetric procedure for the next to your last pregnancy? ______________________ (Ksh)

All in all, were you satisfied with the services that you received during your stay in the delivery ward?

	Not at all	To a small extend	To some extend	To a large extend	To a very large extend	Not sure
45. For the last pregnancy:	○	○	○	○	○	○
46. For the next to the last pregnancy:	○	○	○	○	○	○

How long did you wait before being seen by a provider?

	0 - 14 min	15 - 29 min	30 - 44 min	45 - 59 min	1 hr or longer
47. For the last pregnancy:	○	○	○	○	○
48. For the next to the last pregnancy	○	○	○	○	○

49. For your most recent pregnancy, how many people were present in the delivery room? ______________________

On average rank the confidence that you had in these following health personnel's professional competence:

	Not at all	To a small extend	To some extend	To a large extend	To a very large extend	Not sure
50. Nurses	○	○	○	○	○	○
51. KRNAs	○	○	○	○	○	○
52. Doctors	○	○	○	○	○	○

What happened when you received anesthesia:

	Yes	No
53. Did you go to sleep?	◯	◯
54. Did they stick a needle in your back and you were awake?	◯	◯

55. Are you familiar with the cost of C/S?
◯ Yes
◯ No

56. Is the total cost equal to:
◯ 1 day's wage
◯ 1 week's wage
◯ 1 month's wage
◯ 3 month's wage
◯ 6 month's wage
◯ 12 month's wage

57. Do you think improvements could be made in service delivery in this facility?
◯ Yes
◯ No

57. a. If yes, which ones?

____

58. Would you return to this facility for a C/S for your next child?
◯ Yes
◯ No

58. a. Why?

____

59. If you had a non-obstetric emergency, would you come to this facility?
◯ Yes
◯ No

60. Did anyone give you a chance to give feedback about your experience?
◯ Yes
◯ No

60.a. If Not, would that be helpful to you?
◯ Yes
◯ No

60. b. Why?

____

61. Have you used the services of a Traditional Birth Attendant (TBA)?
◯ Yes
◯ No

61.a. If you have used the services of a Traditional Birth Attendant:

	Not at all	To a small extent	To some extent	To a large extent	To a very large extent	not sure
How satisfied were you with the services you received from the TBA?	◯	◯	◯	◯	◯	◯

	Very Negative	Negative	Neutral	Positive	Very Positive
62. Rank the perception of the community on obstetric care at this health facility:	◯	◯	◯	◯	◯

63. Did you know that there are newly trained anesthesia nurses who have been employed by this hospital and that they help with safe C/S?

◯ Yes
◯ No

64. Does being aware of this make you more comfortable going to the Hospital for surgery?

◯ Yes
◯ No
