# Supplementary material for: Factors influencing mothers’ decisions regarding obstetrical care in Western Kenya: a mixed-methods study
Source: BMC Womens Health. 2021 May 19;21:210. doi: 10.1186/s12905-021-01355-9 (PMC8136230; doi:10.1186/s12905-021-01355-9)
Supplement: Supplementary file 2 — Additional file 2: Appendix 2. Focus group guide. This tool, containing open-ended scripted questions, was used by the focus group moderator to help guide the focus group interviews and discussion. The focus group sessions were held in English, Swahili, or Luo, depending on the language preferred by the participants. [file 12905_2021_1355_MOESM2_ESM.pdf]

ImPACT AFRICA

Improving Perioperative Anesthesia Care and Training in Africa

CONSENT FORM

COMMUNITY FOCUSED GROUP DISCUSSION

Evaluation of the KRNA training program on obstetric outcomes-pilot study

I want to thank you for taking your time to meet/speak with me today. My name is _______________________. I am working with Maseno University, AIC Kijabe and Vanderbilt University. We are conducting a survey about health and specifically obstetric surgery services in Siaya County and all over Kenya. The information we collect will help the KRNA training program in Kijabe and the MOH to plan health service. You were selected to be part of this survey group discussion.

Remember, you don’t have to talk about anything you don’t want to and you may end the interview at any time. The interview will not take too much time (approximately 90 minutes). Everything will be handled in confidentiality and we will use personal identifiers to ensure anonymity. If you agree, I would like to ask you some questions about the topics we discussed and record your responses. May I proceed with the first question?

(*Proceed with the guide that includes topics to be covered and questions that may be helpful in facilitating the interview. You do NOT have to ask all the questions or follow the order given in the guide*.)

……………………………………… ………………………………………………

Signature of the interviewer Date (DD/MM/YYYY)

Community Focus Group Questions

1. What is the average number of pregnancies in this community per year?
2. What is the perception in this community of people delivering at the Hospital?

3. Why do women choose not to deliver at the hospital?
   >>>*Probe1: Lack of trust in the health provider, long distance to get to the closest health facility, etc.*
   >>>*Probe2: Has this changed at all?*

4. How about Traditional Birth Attendants (TBA); what is the perception of people delivering with a TBA?

5. What is the perception of C-section as opposed to normal delivery in this community?
   >>>>>*Probe 1: Do people think it is safe?*
   >>>>>*Probe2: Do you think it is safe?*

6. Describe the changes in the past year or two that have taken place with surgery and anesthesia care in this community?
   >>>*Probe1: How were these cases taken care of before?*Has this changed?
   >>>>*Probe2: Have the problems from the past been addressed?*

7. Give us an estimate of what the cost of obstetric surgery would be equal based upon your monthly income?
   >>*Probe1: a tenth? a third? half? Of your monthly income or all your monthly income?*
   >>>> *Probe2*: Would you be willing to pay it?
   >>>> *Probe2*: Can people in this community on average afford it?

8. How about the transportation? Can you get transport to the facility if you need help when pregnant?
   >>>>*Probe: Is this a serious issue or not a big deal to get TO the facility?*

9. How many women would you know about who died in the community per year because she could not get the healthcare facility?
   >>>Give me your best estimate

10. What are the most important changes that should be done in the maternity services?
    >>>*Probe1: What are the community solutions to mothers needing safe surgery?*
    >>> *Probe2*: *What the actions that the community is taking to make healthcare more affordable?*
